# Isolated Ophthalmoplegia: An Uncommon Presentation of Anti-glutamic Acid Decarboxylase 65 (Anti-GAD65) Neurological Syndrome

**DOI:** 10.7759/cureus.75375

**Published:** 2024-12-09

**Authors:** Roberto A Cruz, Oscar Gutierrez Treviño

**Affiliations:** 1 Neurology, Neurology Institute, Doctors Hospital at Renaissance, McAllen, USA; 2 Neurology, Tecnológico de Monterrey, Monterrey, MEX

**Keywords:** anti-gad antibodies, anti-glutamic acid decarboxylase, auto immune, auto-immune neuropathy, cranial neuropathy, ophthalmoplegia syndrome

## Abstract

Isolated ophthalmoplegia as an anti-glutamic acid decarboxylase 65 (anti-GAD65) antibody-associated neurological syndrome is rare. We present a case of a 22-year-old pregnant Hispanic female patient who presented initially with a left oculomotor nerve palsy following an emergency department (ED) visit for migraine headache. Brain imaging was done with no important findings. The patient was managed with pyridostigmine as a myasthenia gravis diagnosis was suspected but no response was seen. Left oculomotor palsy went on to improve gradually with a course of oral steroids; however, on her third-month follow-up, the patient developed a worsening diplopia episode revealing a new left abducens nerve palsy followed by right oculomotor and trochlear nerve palsies in a period of less than one month. An autoimmune encephalitis panel was made which came back positive for anti-GAD65. A recommendation was made to start intravenous immunoglobulin (IVIG); however, the insurance company only approved mycophenolate mofetil which went on to mitigate the aforementioned palsies. Our case supports the efficacy of steroids and mycophenolate mofetil as reasonable immunomodulators for GAD65-associated neurological syndromes. However, further research is needed to determine the best appropriate treatment approach.

## Introduction

Anti-glutamic acid decarboxylase 65 (anti-GAD65) antibody is known to be present in several endocrine autoimmune disorders like diabetes mellitus and thyroid disease [1]. Several neurologic syndromes have been linked to this antibody as well, such as autoimmune encephalitis presenting as epilepsy, Miller Fisher syndrome (MFS), and stiff-person syndrome (SPS) with some of these reporting ophthalmoplegia [2-6]. A case of isolated ophthalmoplegia associated with positive anti-GAD antibody titers was reported in 2021; however, this patient developed global ataxia later in the disease course [7]. To our knowledge, as of today, there are no reports of isolated ophthalmoplegia with positive GAD65 autoantibody titers. Here we describe a case of isolated atypical ophthalmoplegia with positive serum anti-GAD65 antibodies. 

## Case presentation

A 22-year-old right-handed Hispanic female patient was admitted to the hospital after a worsening episode of what initially was thought to be a typical left-sided migraine without aura with associated hypertension. At the time of admission, the patient was pregnant and at 35 weeks gestation. She was treated in the emergency department (ED) as a newly diagnosed preeclampsia for which delivery was made. During this admission, the patient developed a left oculomotor nerve paralysis (CNIII) for which a neurology consultation was requested. Interestingly, the patient had an episode of peripheral facial nerve paralysis (CNVII) that was thought to be Bell’s palsy when she was 13 years of age which self-resolved. She did have a known diagnosis of migraine headaches treated only with over-the-counter analgesics.

**Figure 1:**
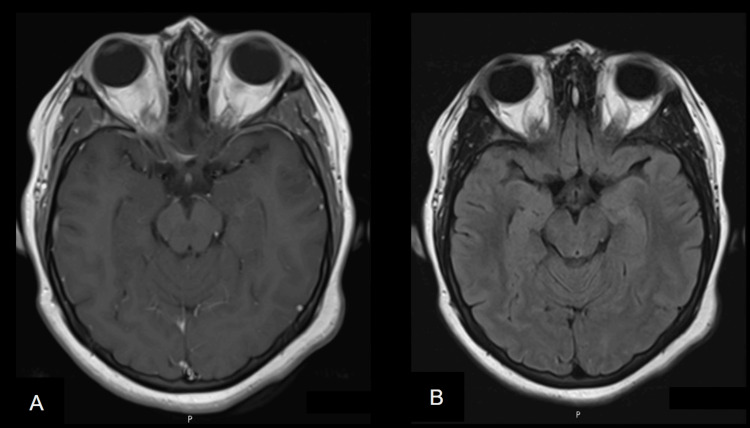
A) T1-weighted MRI post-gadolinium injection and B) Axial FLAIR MRI with normal-appearing brain parenchyma and midbrain. FLAIR MRI: Fluid-attenuated inversion recovery magnetic resonance imaging

**Figure 2:**
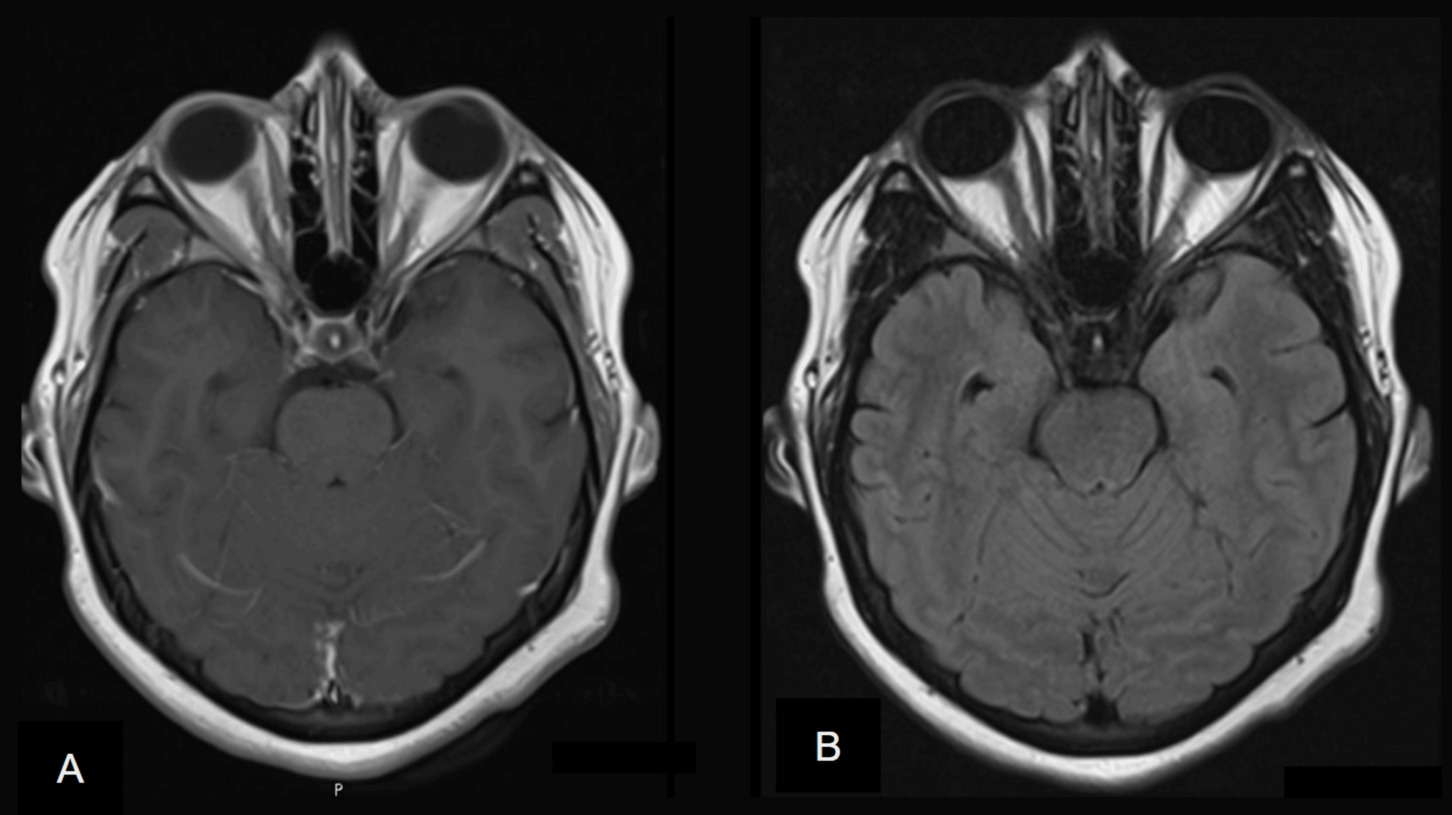
A) T1-weighted MRI post-gadolinium injection and B) Axial FLAIR MRI with normal-appearing optic nerves, extraocular muscles, brain parenchyma, and pons. FLAIR MRI: Fluid-attenuated inversion recovery magnetic resonance imaging

**Figure 3:**
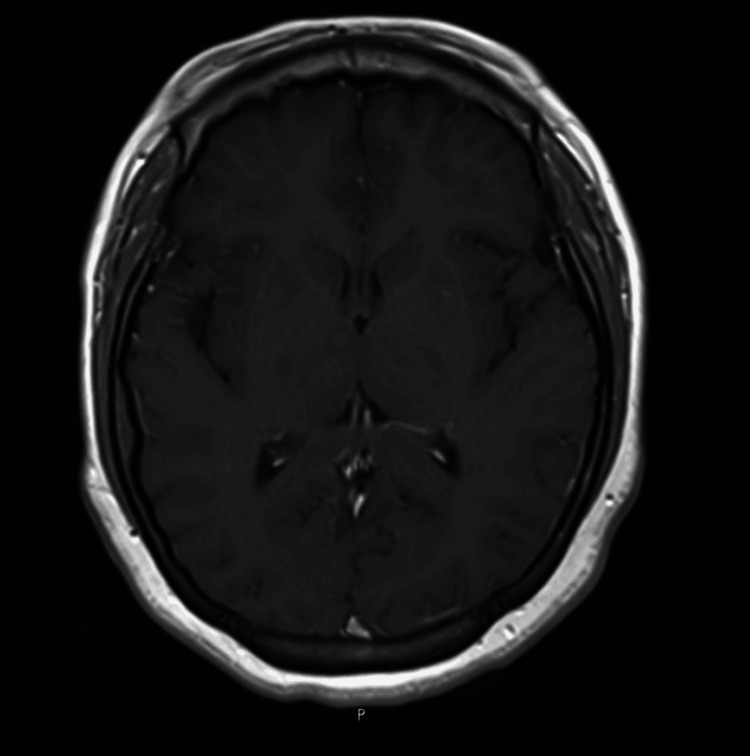
T1-weighted MRI showing normal lateral ventricles, basal nuclei, thalami, and normal-appearing white and gray matter. FLAIR MRI: Fluid-attenuated inversion recovery magnetic resonance imaging

**Figure 4:**
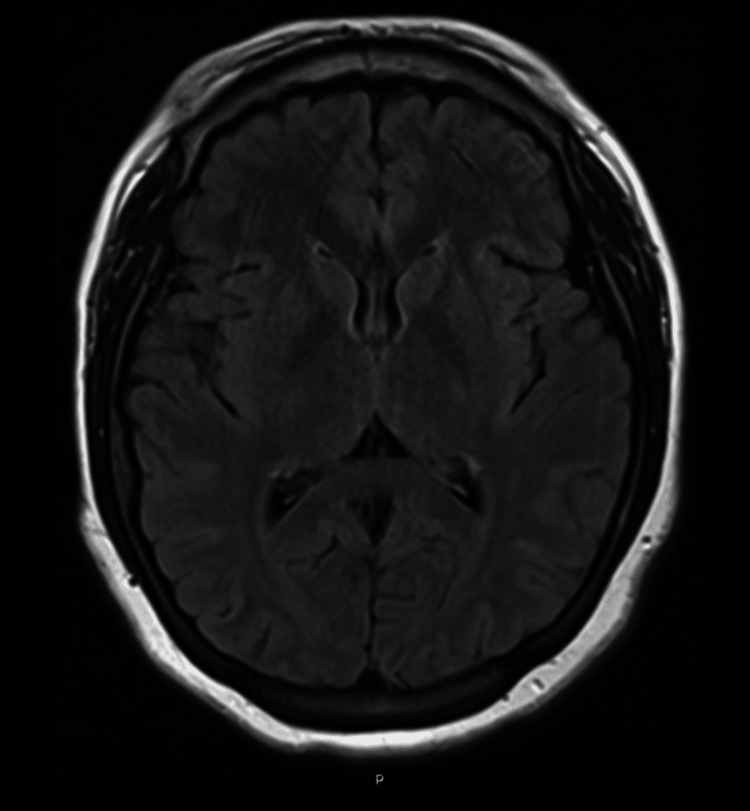
Axial FLAIR MRI with normal-appearing cortical and subcortical brain parenchyma as well as normal-appearing ventricles. FLAIR MRI: Fluid-attenuated inversion recovery magnetic resonance imaging

On neurological examination, the patient exhibited a left oculomotor (CNIII) paralysis with evident exotropia with an unaffected pupil, examination in the right eye was normal without cranial nerve abnormalities. No motor, sensory, balance, or gait abnormalities were found on physical exam. A computed tomography (CT) without contrast of the head was normal as well as magnetic resonance imaging (MRI) and angiogram of the brain (Figures 1-4). Myasthenia gravis was suspected due to its high prevalence in this age group and its frequent presentation with isolated ocular symptoms. Consequently, acetylcholine receptor (AChR) antibody testing was obtained, and a trial of pyridostigmine 60 mg three times daily along with prednisone 20 mg daily, followed by a two-week taper, was initiated. Due to chronic migraines, the patient was also given topiramate 50 mg daily as a maintenance therapy. AChR antibodies for myasthenia gravis and anti-GQ1B for MFS later came back negative. Pyridostigmine was discontinued after six weeks as there was no response to treatment.

Left oculomotor (CNIII) paralysis improved after steroids were initiated; however, on her third-month outpatient follow-up visit, she reported a recurrence of diplopia. Interestingly, physical examination now revealed a new left abducens nerve (CNVI) paralysis which was confirmed by ophthalmology. An autoimmune serology panel (Table 1), encephalopathy autoimmune/paraneoplastic serum panel (Table 2), and cerebrospinal fluid (CSF) analysis (Table 3) were made, and the patient was started on a new course of oral prednisone. This regimen improved left abducens nerve (CNVI) paralysis; however, the patient went on to develop an oculomotor (CNIII) and trochlear (CNIV) nerve palsy on the right eye with the left eye being normal a month later; this was noted at neuro-optometry evaluation. Anti-GAD65 came out positive in serum with a low titer of 0.05 nmol/L; CSF protein, glucose, and white blood cells were all within normal limits as seen in Table 3. 

**Table 1:** Autoimmune serology panel results.

Autoimmune Serology Panel		
	Results	Reference Ranges
ANA, Serum	0.1 Ratio	<0.7 Ratio
dsDNA	1.5 IU/ml	<10 IU/ml
SS-A/Ro Antibody	<0.4 U/ml	<7 U/ml
SS-B/La Antibody	<0.4 U/ml	<7 U/ml
Rheumatoid Factor Quantitative	<10.0 IU/ml	<10 IU/ml
ACE, Serum	14 U/L	9-67 U/L

**Table 2:** Autoimmune encephalitis and paraneoplastic syndrome serum panel results.

Encephalopathy, Autoimmune/Paraneoplastic Serum Panel		
	Results	Reference Ranges
GAD65 Ab Assay, S	0.05 nmol/L	0.02 nmol/L
Amphiphysin Ab, S	Negative	Negative
AGNA-1, S	Negative	Negative
ANNA-1,2 and 3, S	Negative	Negative
CASPR2-IgG CBA, S	Negative	Negative
CRMP-5-IgG, S	Negative	Negative
DPPX Ab IFA, S	Negative	Negative
GABA-B-R Ab CBA, S	Negative	Negative
GFAP IFA, S	Negative	Negative
IgLON5 IFA, S	Negative	Negative
LGI1-IgG CBA, S	Negative	Negative
mGluR1 Ab IFA, S	Negative	Negative
Neurochondrin IFA, S	Negative	Negative
NIF IFA, S	Negative	Negative
NMDA-R Ab CBA, S	Negative	Negative

**Table 3:** Cerebrospinal fluid analysis results.

CSF Analysis		
	Results	Reference Ranges
Appearance	Clear	Clear
Color	Colorless	Colorless
Protein CSF	22 mg/dl	15-45 mg/dl
Glucose CSF	60 mg/dl	40-70 mg/dl
RBC CSF	0 / HPF	0 / HPF
WBC CSF	1 / HPF	0 / HPF
Segmented CSF	0 / HPF	0 / HPF
Lymphocytes CSF	0 / HPF	0 / HPF
Eosinophils CSF	0 / HPF	0 / HPF

Intravenous immunoglobulin (IVIG) was recommended; however, despite significant efforts, her health insurance did not approve this treatment, for which mycophenolate mofetil 500 mg twice a day was started instead. At the six-month follow-up, the patient remained clinically stable with this pharmacological regimen without any new cranial neuropathy, or any other neurological symptom attributed to anti-GAD65. 

## Discussion

Glutamic acid decarboxylase (GAD) is a GABA synthesis pathway enzyme that is known to be present intracellularly around synaptic vesicles in GABA-secreting neurons and other types of cells [8]. Autoantibodies against GAD65 kilodalton subunit have been found in several non-neurologic and neurologic diseases such as diabetes mellitus type 1, thyroid disease, SPS, limbic encephalitis, and cerebellar ataxias [2,7-9]. Taking into account GAD's catalytic role, having autoantibodies against it would theoretically affect GABA-producing neurons and their respective synthetic function. In fact, it has been demonstrated that their presence causes GABAergic neuron dysfunction. To build up on the same hypothesis, GABA-enhancing drugs such as vigabatrin and baclofen have improved symptoms in patients with SPS, which confirms the impairment of neurotransmission secondary to a neurotransmitter deficiency [10].

Atypical cases of GQ1b seronegative MFS and elevated titers of anti-GAD65 antibodies have been reported; however, these patients presented the classic symptomatology of ophthalmoplegia, ataxia, and areflexia. Intravenous gamma globulin infusions appeared to improve the symptoms in some cases while only oral steroids ameliorated symptoms in one case. A decline of anti-GAD65 titers was seen in two of the cases after their respective treatment regimen [3-5]. Gupta and Liu reported an atypical case of MFS on a patient presenting with diplopia who was found to have progressive internal and external ophthalmoplegia with frequent fluctuations in pupillary diameter. Serum testing revealed positive titers of anti-GQ1b and anti-GAD antibodies, and the patient was treated with a course of IVIG and IV steroids with mild symptom improvement. This patient did not go on to present any of the typical symptoms of MFS such as ataxia or areflexia, making this clinical presentation a possible variant of the syndrome [11]. 

Belem et al. described the first case of isolated ophthalmoplegia in a patient with positive serum anti-GAD65 antibodies. This patient presented vertical misalignment and hypertropia of the right eye which upon individual eye examination was found to be secondary to tonic vertical deviation of the eye. Later on the disease course, the patient developed global ataxia, clinically confirmed with a scale for assessment and rating of ataxia (SARA) score of 11. In the same manner that the previously presented cases were treated, this patient was given methylprednisolone for five days with mild symptom improvement. One month later, with a treatment course of repeated methylprednisolone along with IVIG for five days, significant sign and symptom improvement was seen with complete resolution after one week with continuous absence of symptoms four years after the initial presentation [7]. To our knowledge, there are currently no reported cases of isolated ophthalmoplegia with positive anti-GAD65 antibody titers. This case shows that anti-GAD-associated neurological diseases are still ambiguous and can present as a sole symptom without a strict association to a particular syndrome such as SPS or MFS. On the other hand, it may also mean that these syndromes have a wide variety of presentations that may sometimes lead to underdiagnosis of them. 

## Conclusions

Taking into consideration the different treatment approaches within our case and the other reported cases, IVIG has been demonstrated to be effective in improving signs and symptoms. Oral and intravenous steroids are also a reasonable approach in the acute setting; moreover, mycophenolate mofetil in our case was an alternative treatment that appeared to help maintain good control of symptoms. More research is needed in order to determine the best appropriate treatment for anti-GAD65-associated neurological syndromes. 
